# Trend in 167 cases of minors witnessing violence: The role played by COVID-19 pandemic

**DOI:** 10.3389/fped.2022.949922

**Published:** 2022-10-05

**Authors:** Martina Focardi, Simone Grassi, Silvia Raddi, Maria Elena Rosati, Francesca Cazzato, Paola D’Onofrio, Vittoria Doretti, Ilenia Bianchi, Giuseppe Vetrugno, Antonio Oliva, Vilma Pinchi

**Affiliations:** ^1^ Department of Health Science, University of Florence, Florence, Italy; ^2^ Legal Medicine, Department of Health Surveillance and Bioethics, Università Cattolica del Sacro Cuore, Rome, Italy; ^3^ Responsible for the Unit of the Rose Code, Area of Emergency Rooms of Careggi Hospital, Florence, Italy; ^4^ Head of Department Health Promotion and Health Ethics, Azienda USL Toscana Sud Est, Careggi Hospital, Florence, Italy; ^5^ Department of Law, University of Macerata, Macerata, Italy

**Keywords:** abuse, witnessed violence, violence, children, COVID-19

## Abstract

**Background:**

There currently is no evidence that COVID-19 has had an impact on the rates of psychological abuses occurring when a minor witnesses interpersonal violence.

**Aim:**

Our aim was to describe the accesses of the last four years to the Emergency Department of a tertiary hospital (Careggi University Hospital—Florence, Italy) due to this issue and then to evaluate whether the COVID-19 has had an impact on this trend.

**Methods:**

We collected data regarding cases of abuse in which at least a minor had reportedly witnessed the event. Medical records stored between January 1, 2018 to January 1, 2022 were analyzed, extracting sex, age and nationality of the victim; sex of the perpetrator and relationship with the victim; known previous episodes of abuse in the medical history of the victim; setting of the abuse (domestic vs. non-domestic); type of abuse (physical, psychological, sexual); whether the perpetrator was under the influence of alcohol/drugs; whether the victim was hospitalized; prognosis of the victim; number, relation with the victim and involvement in the abuse (as co-victim) of the minor(s) who witnessed the abuse.

**Results:**

A total of 167 eligible cases were registered. 69% of victims had previous episodes of abuse. The perpetrators were all known and mainly males (96%).The abuses were mainly domestic (79%). In 74% of the cases only a type of violence was perpetrated. In 12% of the cases, the minors were also victims of physical abuse. No statistically significant relationships were found between the start of the COVID-19 pandemic and the changes in the number of cases of domestic abuse (*p* = 0.07), physical abuse (*p* = 0.62), psychological abuse (*p* = 0.83) or sexual abuse (*p* = 0.88). However, during the institutional lockdown in Italy (March-May 2022) only two cases occurred – a number that did not allow period-specific statistical inference.

**Conclusions:**

Empowering the hospital policies specifically aimed at identifying and protecting the victims of violence/witnessed violence remains a critical goal from both a public health and medico-legal point of view.

## Introduction

Child abuse and domestic violence represent serious public health issues associated with severe long-term physical, social and mental health outcomes along with relevant economic burden (the only cases of child sexual abuse in the United States entail lifetime economic costs for 9.3 billion $) (1–6). There are several kinds of abuse, like physical, psychological and sexual abuse (1, 7).

Witnessing violence is recognized as a form of psychological child abuse (8–11). Indeed, even only the awareness of potential violence between caregivers can be experienced by a minor in an extremely stressful fashion (10). From a legal point of view, in European Union, treaties (Istanbul Convention) and national laws (e.g., in Italy, Law n. 69/2019) recognize minors who witness violence as victims of abuse, stress the importance of combining repression and prevention and regulate mandatory reporting in these cases.

Children who witness violence – especially in case of recurrent episodes –tend to develop impaired attachment to primary caregivers and are exposed to significant physical, social and psychological issues like post-traumatic stress disorder and suicide (1, 2, 12–16). The particular vulnerability of the children is due to their scarce cognitive ability to distinguish between a threat to an adult and a threat to themselves (1).

The adverse outcomes tend to emerge during adolescence, for instance as anti-social/risky behaviors, since the scarce parental attachment and problems in the domain of externalizing (e.g., aggressivity) can lead to violent behaviors and delinquency (9, 17). On the other hand, minors who start to witness violence during adolescence are exposed to risks similar to younger children. Indeed, adolescents can be impaired in their psychological and emotional normal growth, thus being highly exposed to risks of mental and/or physical disturbances or unhealthy/anti-social/risky behaviors like the use of illicit substances (9, 18–23). Moreover, these minors are at risk of becoming authors of interpersonal violence during adulthood (1, 2, 6).

Among the factors that increase the incidence of abuses in a population, there certainly is the occurrence of disasters as a collective and highly stressful situation (7). COVID-19 pandemic is one of the most significant examples of mass disaster in human history, having imposed radical changes to the society and families from social and behavioral points of view (24). In particular, according to several authors, COVID-19 pandemic – and in particular the subsequent institutional lockdowns – has exposed the victims of domestic violence at higher risks, forcing them to remain with their perpetrators and making it difficult for them to access health/mental care facilities (1, 25). Moreover, the pandemic has increased the risk for the minors to witness domestic violence, since the schools' closure (1, 25).

However, Ferrara et al. underlined that, albeit COVID-19 led to an upward trend in domestic violence, very little attention has been paid to the effect of the pandemic on the incidence of minors witnessing domestic abuse (16).

Our aim was to describe the trend of the last four years in the accesses to the Emergency Department of a tertiary hospital (Careggi University Hospital—Florence, Italy) due to abuses in which at least a minor had reportedly witnessed the event and then to evaluate whether the COVID-19 has had an impact on this trend.

## Materials and methods

We collected data regarding cases of abuse in which at least a minor had reportedly witnessed the event in the specialized section of the Emergency Department of a tertiary hospital (Careggi University Hospital—Florence, Italy). In particular, when a patient reports abuse at the hospital admission, a specific code is assigned and a specialized medical unit is alerted. In cases of abuses witnessed by minors, the information obtained from the patient is recorded and communicated to the police as required by national law.

Medical records stored between January 1, 2018 to January 1, 2022 were analyzed, extracting in anonymous way these data: sex, age and nationality of the victim; sex of the perpetrator and relationship with the victim; known previous episodes of abuse in the medical history of the victim; setting of the abuse (domestic vs. non-domestic); type of abuse as reported by the victim (physical, psychological, sexual); whether the perpetrator was under the influence of alcohol/drugs; whether the victim was hospitalized; prognosis of the victim; number, relation with the victim and involvement in the abuse (as co-victim) of the minor(s) who witnessed the abuse.

Statistical analysis (Pearson's chi-squared test) was performed to evaluate whether the pandemic has had an impact on the relationship between the primary victim and the perpetrator and on the number of cases of domestic abuse, physical abuse, psychological abuse and sexual abuse. In order to perform it, cases were grouped in four different classes: (A) cases occurred in 2018 and 2019; (B) cases occurred in 2020 and 2021; (C) cases occurred in March-May 2020 (the period of the institutional lockdown in Italy); (D) cases occurred in March-May 2019. Variables were compared between class A and class B and between class C and class D.

A *p*-value equal to or lower than 0.05 was set as cut-off for statistical significance. The software used for statistical analysis was IBM SPSS Statistics for Windows, Version 24.0 (IBM Corp., Armonk, NY).

The study was approved by the competent institutional research ethics committee and was performed in accordance with the ethical standards as laid down in the 1964 Declaration of Helsinki and its later amendments or comparable ethical standards.

## Results

**Figure 1:**
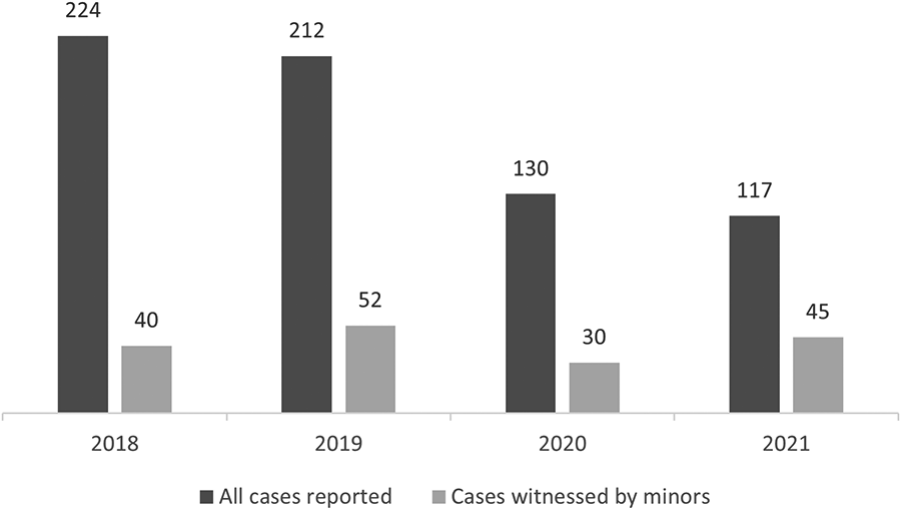
Admissions due to abuse in general and to abuse reportedly witnessed by at least a minor.

683 cases of abuse in general and 167 victims of abuse witnessed by at least a minor were admitted to the specialized section of the Emergency Department during the study period (Figure 1).

Regarding the primary victims targeted by the perpetrator (Table 1), the 96% (160) of them were females and only the 4% (7) males; only the 1% (2) of them were minors; the 49% (81) were not of Italian nationality. Moreover, the 69% (116) of victims had previous episodes of abuse in their medical history. In 3 out of 167 (2%) cases the victim was hospitalized. The prognosis ranged between 0 and 40 days, with a mean value of 7 days and a median of 6 days.

**Table 1:** Primary victims (Panel A) and perpetrators (Panel B) main characteristics.

**Panel A: primary victims**		
Sex	Male	96% (160)
	Female	4% (7)
Age	Minor	1% (2)
	Adult	99% (165)
Nationality	Italian	51% (86)
	Others	49% (81)
Previous episodes of abuse	Yes	69% (116)
	No	31% (51)
Hospitalized	Yes	2% (3)
	No	98% (164)
**Panel B: perpetrators**		
Sex	Male	96% (161)
	Female	4% (6)
Crime committed under alcohol/drug influence	Yes	5% (9)
	No	95% (158)

The perpetrators were all known and mainly of male sex (96%–161%), and only in 9 cases (5%) the perpetrator committed the violence under the influence of alcohol and/or drugs (Table 1).

**Figure 2:**
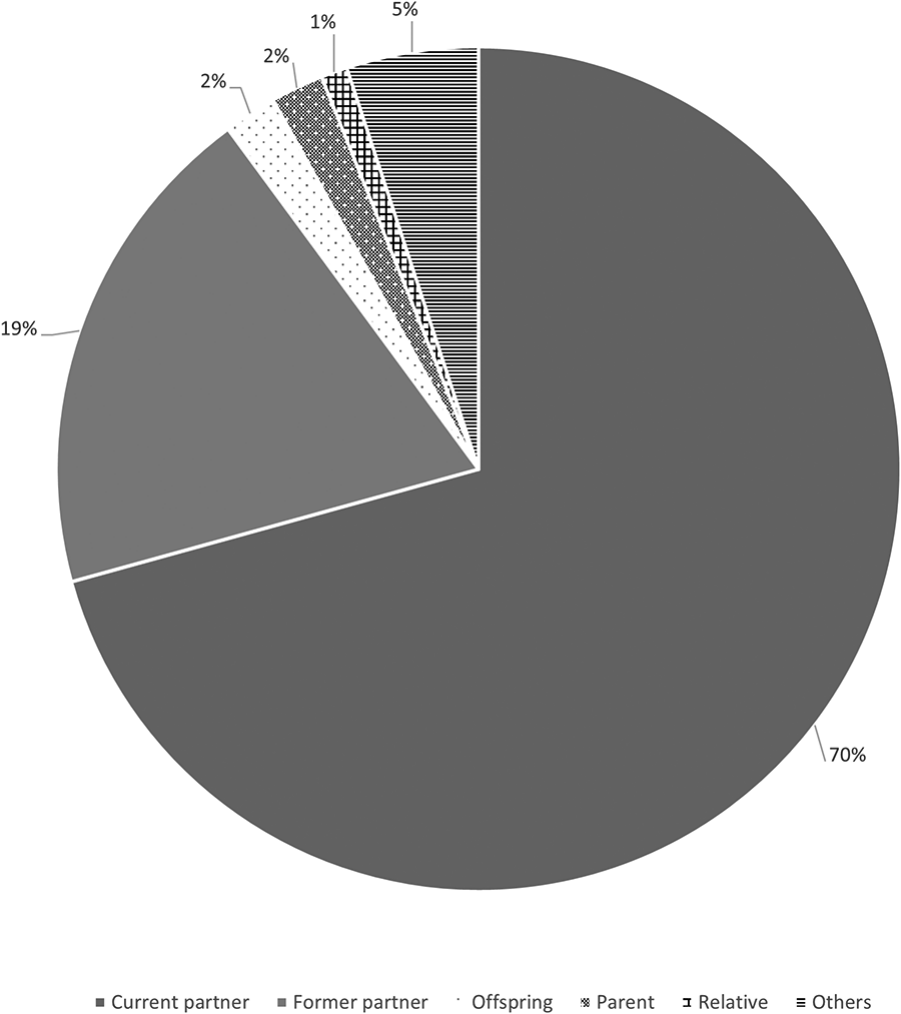
Relationship between the primary victim and the perpetrator.

The perpetrator was the partner at the time of the crime, the former partner, the son, the parent or a relative of the primary victim in, respectively, 117 (70%), 32 (19%), 4 (2%), 4 (2%) and 2 (1%) cases (Figure 2). Regarding these variables, no statistically significant relationship difference between class A and class B (*p* = 0.64).

The abuse was perpetrated more frequently in the domestic setting (132 cases – 79% vs. 35 cases −21%) (Table 2).

**Table 2:** Abuse circumstances.

Setting	Domestic	79% (132)	
	Others	21% (35)	
Type of violence	Physical	114 (68%)	Total 123 (74%)
	Psychological	9 (6%)	
	Physical and psychological	42 (25%)	Total 44 (26%)
	Physical and sexual	1 (0.5%)	
	Physical, psychological and Sexual	1 (0.5%)	
How many minors witnessed	Only one	121 (72%)	
	More than one	46 (28%)	
Child both witnessed and primary victims		20 (12%)	

In 123 (74%) cases only a type of violence was perpetrated: 114 (68%) cases consisted in physical abuses and 9 (6%) in psychological ones. In the remaining 44 (26%) cases, at least two kinds of abuse co-occurred: in 42 (25%) physical and psychological; in a case physical and sexual; in a case physical, psychological and sexual (Table 2).

In 121 (72%) cases, only a child witnessed the abuse, while in the remaining 46 (28%) cases more children assisted to violence. In 12% (20) of the cases, the minors not only witnessed the violence towards other people but were also primary victims of physical abuse (Table 2).

**Figure 3:**
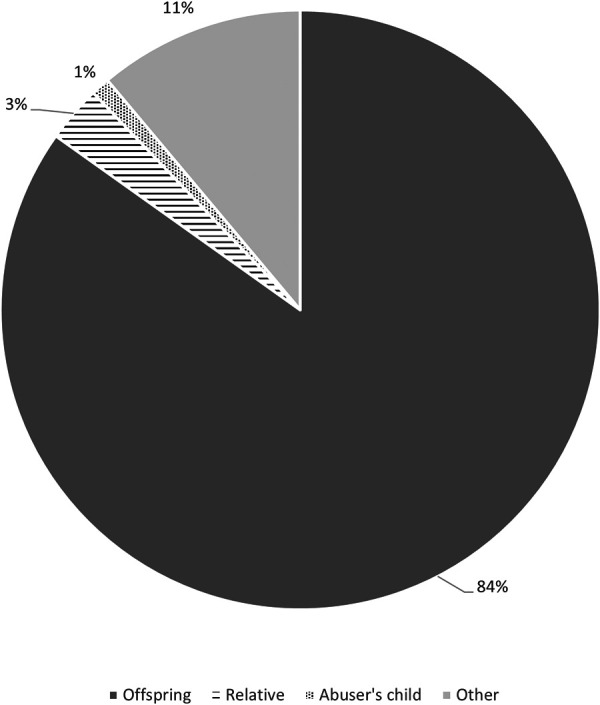
Relationship between the minor witnessing the abuse and the primary victim.

The minor was a child or a relative of the primary victim, respectively, in 141 (84%) and 5 (3%) cases, and a child of the perpetrator in 2 (1%) cases (Figure 3).

In order to perform statistical analysis, cases were grouped in four different classes, as described in the previous section.

First, statistical analysis was performed comparing class A and class B (Table 3).

**Table 3:** Statistical comparison between cases occurred in 2018 and 2019 (A) and cases occurred in 2020 and 2021 (B).

Class	Period (years)	Cases (total no.)	Domestic setting	Physical abuses	Psychological abuses	Sexual abuse
A	2018–2019	92	68	82	28	1
B	2020–2021	75	64	65	24	1
*p*-value			0.07	0.62	0.83	0.88

Class A consisted in 92 cases, of which 68 occurred in the domestic setting, 82 were physical abuses, 28 psychological abuses and a single case of sexual abuse.

Class B consisted in 75 cases, of which 64 occurred in the domestic setting, 65 were physical abuses, 24 psychological abuses and a single case of sexual abuse.

No statistically significant relationships were found between the start of the COVID-19 pandemic and the changes in the number of cases of domestic abuse (*p *= 0.07), physical abuse (*p* = 0.62), psychological abuse (*p* = 0.83) or sexual abuse (*p* = 0.88).

Statistical analysis on the comparison between class C and class D was not performed because, while class D consisted in 11 cases, class C consisted in only two cases (both of them were male perpetrators who perpetrated physical and psychological violence against their partners in the domestic setting).

## Discussion

The research goal was to describe the trend of the last four years in the accesses to the Emergency Department of our tertiary hospital due to abuse in which at least a minor had reportedly witnessed the event and, then, to evaluate whether the COVID-19 has had an impact on this trend.

To the best of our knowledge, it is the first original research paper to describe this phenomenon focusing on the minors who witness violence and to try to make inference on the possible influence of the pandemic on it.

The role of the COVID-19 is of great scientific interest because, albeit – as said – disasters usually led to an increase in violence, the pandemic led to isolation and reduced the access to healthcare. Indeed, Viero et al. reported a downward trend in the access to the services dedicated to the victims of violence in an Italian center during the COVID-19 lockdown (7). Despite this, in 2022 Anastasia et al. performed an observational cross-sectional study on 212 mother–child dyads from February 2020 to January 2021, finding a 43.9% prevalence of intimate partner violence among the mothers at the pediatric Emergency Department (and thus inferring that there was a similar prevalence of children exposed to violence) (11).

In our study, only 30 cases were reported in 2020 (the year in which the access to health services was more difficult), while in 2021 the number of the cases (45) was even higher than that of 2018. The statistical analysis performed to compare the pre-pandemic period with the last two years failed to find a statistically significant explanation for these changes. However, if only the period corresponding to the institutional lockdown (March–May 2020) is considered, only two cases occurred, regarding women who were victims of physical and psychological abuse perpetrated by their partners in the domestic setting. This number suggests an influence of the pandemic even if, at the same time, makes impossible to perform a statistical comparison.

As said, the setting of the abuse is extremely relevant for the minor who witnesses violence. Indeed, more than 50% of the cases of physical or psychological abuse of minors is reportedly perpetrated by household members (26). In accordance with this evidence, the striking majority of our cases occurred in the domestic setting (79%).

The incidence of intimate partner violence has been reported to be higher among ethnic minorities and in cases of acute/chronic use of alcohol and/or drugs (1, 17, 27). Instead, in our cases the 49% of the victims belonged to an ethnic minority and only in the 5% a state of acute intoxication of the perpetrator was reported.

In the 12% of our cases, the child was not only a victim of the psychological abuse related to having witnessed violence but was also a victim of physical abuse. Minor abuse and minor exposure to violence frequently co-occur, especially when the violence is domestic (28). Moreover, about the 71, the 56% and 51% of the children who witness intimate partner violence are also victim of, respectively, sexual, physical and emotional abuse (29). In these cases of co-occurrence of different kinds of abuse, the term polyvictimization should be used. Polyvictimization represents a particularly severe issue, because it is known to relate to more severe outcomes for the minor (30).

Another important evidence we found is that the 69% of the cases consisted in recurrent episodes of abuse. Indeed, abusers tend to re-perpetrate violence (1, 6). Moreover, (any form of) child abuse is a transgenerational problem, since abused minors are exposed to a higher risk of becoming abusers during the adulthood (8, 9). The relationship between recurrence of the abuse and risk of becoming a abuser is due to the fact that, according to the social learning theory, the minor who witnesses frequent episodes of violence develops the idea that using violence in the interpersonal relationships is normal and thus is prone to imitate the experienced behavior (9).

Since – as said – abuse entails a high risk of adverse outcomes for the victim and the risk of transforming the victim into a future perpetrator, exposure of a minor to violence has been included in worldwide mandatory reporting legislation (10).

Instead, from a public health point of view, several authors advocated an empowerment of the interventions targeted at enhancing the help-seeking behaviors and the identification/protection of the cases of interest, with particular regard to the high-risk categories (like institutionalized minors) (1, 7).

In particular, specialized health services dedicated to the victims of violence and to the minors who witness violence are crucial parts of a public health strategy aimed at contrasting the social/legal/economic implications of abuse and its long-term health outcomes (11). Indeed, this issue is often misrecognized and underaddressed by non-specialized healthcare professionals, leading to a failure to implement early professional-driven interventions for secondary prevention (e.g., counselling, cognitive behavioral therapy) and to report the crime(s) to the competent public authorities (7, 8, 10, 31–36). Indeed, the professionals operating in healthcare often do not have a clear idea of what should be considered reportable and they tend to avoid to report the exposure of children to interparental violence because of the fear of a further victimization of the abused parent (10). The two most important interventions to address these issues are represented by a rigorous education of the personnel and the inclusion of experts in legal/forensic medicine into the multidisciplinary teams that visit the cases of abuse (6).

A professional-driven approach to the identification of these cases is also needed because minors can be impacted in disclosing the witnessed episode, since feelings of isolation, shame, fear and guilt are very common (9, 28). As said, early identification of abused minors can limit or avoid negative outcomes enhancing their coping skills and allowing the development of a positive relationship with caring, non-abusive adults (28).

In particular, as stressed by Offidani et al., these specialized services must be provided by Emergency Departments (as in our institution), in order to allow an early identification of these cases of interest and thus to address their specific medical, social and legal needs in a proper and effective fashion (31).

Finally, as suggested by our data, pandemic can influence the accesses to these centers, and thus the adoption of evidence-based interventions to enhance the safety of the healthcare environment must be implemented to encourage the victims of violence to seek assistance and to grant them, especially in cases of domestic violence, a safe environment in which they can live.

## Conclusion

Despite mass disasters usually leading to an upward trend in abuses (particularly referring to domestic abuses), there is currently no evidence that COVID-19 has had an impact on the rates of the specific form of psychological abuse occurring when a minor witnesses interpersonal violence. According to our cases, the most likely explanation is given by the extremely few cases reported during the phase of the institutional lockdown. This fact represents an issue because – as shown by our cases – most of the abuses of interest is to be classified as intimate partner violence, and during the institutional lockdown both the abused partners and the minors were forced to stay with their perpetrators and, at the same time, tended to avoid accessing to hospitals since the pandemic. However, in order to try to overcome the limitation given by the few reported cases during the institutional lockdown, multicentric studies are needed. That being said, empowering the hospital policies specifically aimed at identifying and protecting the victims of violence and of witnessed violence is a critical goal from both a public health and medico-legal point of view.

## Limitations

The results of our study stress that future research on this matter should be multicentric, needing the combination of the datasets of several centers to obtain an adequate sample for the statistical analysis regarding the specific and relatively short period of the institutional lockdown.

## Data Availability

The original contributions presented in the study are included in the article/Supplementary Material, further inquiries can be directed to the corresponding author/s.
